# On the pathogenesis of penile venous leakage: role of the tunica albuginea

**DOI:** 10.1186/1471-2490-7-14

**Published:** 2007-09-05

**Authors:** Ahmed Shafik, Ismail Shafik, Olfat El Sibai, Ali A Shafik

**Affiliations:** 1 Department of Surgery and Experimental Research, Faculty of Medicine, Cairo University, Cairo, Egypt; 2 Department of Surgery, Faculty of Medicine, Menoufia University, Shebin El-Kom, Egypt

## Abstract

**Background:**

Etiology of venogenic erectile dysfunction is not exactly known. Various pathologic processes were accused but none proved entirely satisfactory. These include presence of large venous channels draining corpora cavernosa, Peyronie's disease, diabetes and structural alterations in fibroblastic components of trabeculae and cavernous smooth muscles. We investigated hypothesis that tunica albuginea atrophy with a resulting subluxation and redundancy effects venous leakage during erection.

**Methods:**

18 patients (mean age 33.6 ± 2.8 SD years) with venogenic erectile dysfunction and 17 volunteers for control (mean age 31.7 ± 2.2 SD years) were studied. Intracorporal pressure was recorded in all subjects; tunica albuginea biopsies were taken from 18 patients and 9 controls and stained with hematoxylin and eosin and Masson's trichrome stains.

**Results:**

In flaccid phase intracorporal pressure recorded a mean of 11.8 ± 0.8 cm H_2_O for control subjects and for patients of 5.2 ± 0.6 cm, while during induced erection recorded 98.4 ± 6.2 and 5.9 ± 0.7 cmH_2_O, respectively. Microscopically, tunica albuginea of controls consisted of circularly-oriented collagen impregnated with elastic fibers. Tunica albuginea of patients showed degenerative and atrophic changes of collagen fibers; elastic fibers were scarce or absent.

**Conclusion:**

Study has shown that during erection intracorporal pressure of patients with venogenic erectile dysfunction was significantly lower than that of controls. Tunica albuginea collagen fibers exhibited degenerative and atrophic changes which presumably lead to tunica albuginea subluxation and floppiness. These tunica albuginea changes seem to explain cause of lowered intracorporal pressure which apparently results from loss of tunica albuginea veno-occlusive mechanism. Causes of tunica albuginea atrophic changes and subluxation need to be studied.

## Background

Many classifications have been proposed for erectile dysfunction (ED) [1]. A common classification of ED, which integrates the various causes of impotence with the understanding of erectile physiology and functional anatomy, comprises psychogenic, neurogenic, arteriogenic, venogenic, and endocrinologic origin [1,2]. Failure of adequate venous occlusion has been proposed as one of the most common causes of venogenic impotence [3]. The cause of veno-occlusive dysfunction is not exactly known. It may result from several possible pathophysiologic processes which include the presence of large venous channels draining the corpora cavernosa, Peyronie's disease, diabetes and structural alterations in the fibroelastic components of the trabeculae, cavernous smooth muscle and endothelium [4-6].

The treatment of venogenic ED (VED) is penile veins ligation if the known less invasive measures such as intracavernous injection of vasodilators fail to induce adequate erection [7]. It seems that these treatment modalities deal with a secondary effect rather than the primary etiological factor of the venogenic ED; therefore the results of treatment are unsatisfactory [7]. We hypothesized that the cause of venous leakage during erection is an atrophy of the tunica albuginea (TA) with a resulting TA subluxation and redundancy. This hypothesis was investigated in the current study.

## Methods

### Subjects

The study comprised 18 patients (mean age of 33.6 ± 2.8 SD years, range 30–36) with VED of a mean duration of 7.4 SD years (4–10). They gave a history of *erectile dysfunction lasting for *a mean of 2.4 ± 0.6 minutes (range 2–3). Clinical examination including neurologic was unremarkable. The endocrine profile was normal. The patients demonstrated a normal response to penile biothesiometry and normal resting penile brachial indices (PBI) as determined by Doppler ultrasound. Nocturnal penile testing established impaired erectile function. Dynamic infusion cavernosometry and cavernosography demonstrated an incompetent veno-occlusive mechanism.

Seventeen subjects were included in the study as controls. Their ages matched the patients' age (mean 31.7 ± 2.2 SD years, range 29–33). Eight of the 17 subjects had a normal penis while seven had a degloved penis and a torn TA resulting from motor car accidents, and two subjects had penile squamous cell carcinoma involving only the skin of the penis and not encroaching on the TA. All of the control subjects gave a history of normal erection. The history and investigations of all of the studied subjects showed that they had no medical conditions nor were under drug regimens that could cause ED. A consent was given by the subjects to be studied after they had been informed about the nature of the study and the investigations to be done for them. The study was approved by the Cairo University Faculty of Medicine Review Board and Ethics Committee.

### Methods

The intracorporal pressure (ICP) of the 18 subjects with VED and 17 control subjects was recorded by an angiocatheter (Gould Inc, Cleveland, OH). The pressure was measured in the flaccid phase and after inducing erection by intracorporal injection of papaverine in a dose of 60 mg. The catheter was introduced into the corpora cavernosa (CC) and connected to pneumohydraulic capillary infusion system (Arndorfer Medical Specialties, Greendale, WI), the pump of which delivered saline solution continuously via the capillary tube at a rate of 0.6 ml/min. The transducer outputs were registered on a rectilinear recorder (Model RS-3400, Gould Inc, Cleveland, OH). Occlusion of the recording orifice produced a pressure elevation rate that was greater than 250 cmH_2_O/s. To ensure reproducibility of the results, the pressure in each individual was measured at least twice and the mean value was calculated.

During operative interference, TA biopsies measuring 0.3 × 0.3 cm were taken from the subjects with VED and from those with traumatically torn TA and penile carcinoma. They were obtained from the TA on the lateral aspect of the penis, and from the same location in patients and controls. The specimens were fixed in 10% buffered formalin, dehydrated in graded alcohol and processed for histologic examination. Tissues were embedded in paraffin, sectioned at 4 μm and then stained with hematoxylin and eosin and Masson's trichrome stains. The latter stains collagen and elastin blue and smooth muscles red. Photomicrographs were obtained, developed and digitized with 16.7 × 10^6 ^colors at a resolution of 1.024 × 768 on a scanner.

The results of the study were analyzed statistically using the SPSS (statistical package for the social sciences, version 10) to determine the analysis of variance (ANOVA) and Student's t test. Values were given as the mean ± SD. Differences assumed significance at p < 0.05.

## Results

The study *was *completed in all the subjects without adverse side effects. The ICP in the flaccid phase of the control subjects (8 normal subjects) recorded a mean of 11.8 ± 0.8 cmH_2_O and of the patients with VED 5.2 ± 0.8 cmH_2_O, while during induced erection for the control subjects a mean of 98.4 ± 6.2 cm H_2_O was registered (table 1). For the patients with VED, erection was maintained for a mean period of 2.4 ± 0.6 min (range 1–3) during which the intracorporal pressure recorded a mean of 65.2 ± 4.8 cmH_2_O; after this period detumescence occurred and the ICP recorded a mean of 5.9 ± 0.7 cmH_2_O (table 1).

**Table 1:** The intracorporal pressure of the 8 control subjects and 18 patients with venogenic erectile dysfunction (VED) in the flaccid and erectile phases^+^

	**Intracorporal pressure**			
	**VED patients**		**Control subjects**	
	**Mean**	**Range**	**Mean**	**Range**

**Flaccid phase**	5.2 ± 0.8*	4–6	11.8 ± 0.8	10–13
**Erectile Phase**				
**1^st ^2.4 min**	65.2 ± 4.8*	52–79	98.4 ± 6.2	92–106
**Later**	5.9 ± 0.7**	5–7		

+ values were given as the mean ± SD

* p < 0.05

** p < 0.001

p values of patients with VED were compared with those of the control subjects

**Figure 1:**
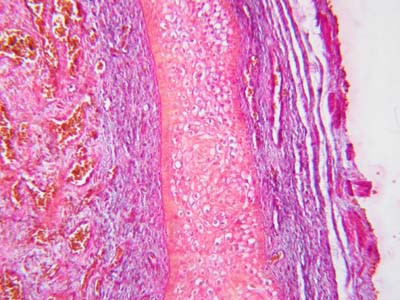
Photomicrograph of tunica albuginea of control subject showing that it consists of collagen impregnated with few elastic fibers (Masson's trichrome stain ×400).

**Figure 2:**
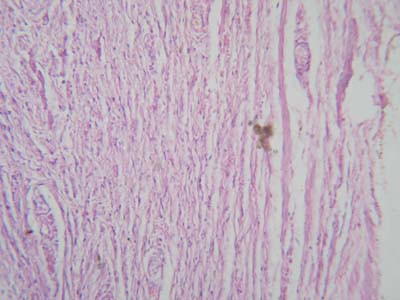
Photomicrograph of tunica albuginea of control subject showing that it consists of collagen fibers arranged circularly and longitudinally (hematoxylin and eosin ×400).

Microscopic study of the TA of 9 of the 17 control subjects (7 with torn TA, 2 with penile squamous cell carcinoma) revealed that it consisted of excessive collagen impregnated with elastic fibers (Fig. 1). The collagen fibers were circularly oriented in all the specimens. In 7 specimens, the circularly arranged collagen fibers were associated with longitudinally arranged collagen fibers (Fig. 2).

**Figure 3:**
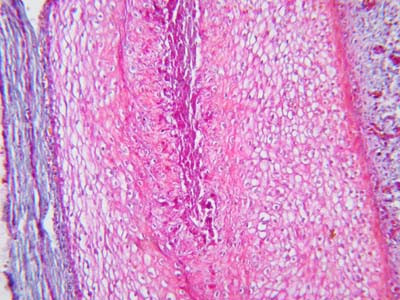
Photomicrograph of tunica albuginea of a patient with venogenic erectile dysfunction showing degeneration and atrophy of the collagen fibers (Masson's trichrome stain ×400).

**Figure 4:**
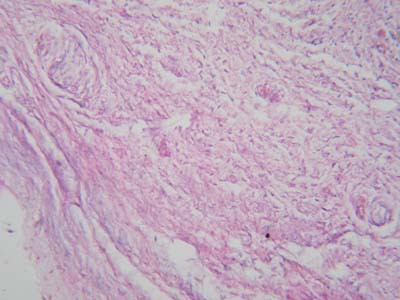
Photomicrograph of tunica albuginea of a patient with venogenic erectile dysfunction showing fragmentation of collagen fibers (hematoxylin and eosin ×400).

Microscopic examination of the biopsies from the 18 patients with VED showed degenerative changes and atrophy of the collagen fibers (Fig. 3). The circular arrangement of the collagen fibers was lost and the fibers were irregularly oriented. The collagen fibers in some specimens were fragmented (Fig. 4) while the elastic fibers were either scarce or completely absent in the specimens

## Discussion

The current study may shed some light on the pathogenesis of VED. The venules that drain the sinusoidal spaces and the smooth muscles of the penis coalesce as they approach the CC periphery and form the subtunical venular plexus [8]. Small veins exit from the plexus through the TA as the emissary veins, and drain into the circumflex veins or directly into the deep dorsal vein. The position of the subtunical venular plexus between the sinusoids and TA allows for their compression and occlusion as the smooth muscle and sinusoids relax and expand against the TA during tumescence [8-11]. This occlusion acts to trap the blood within the penis. The loss of this veno-occlusive function leads to leakage of blood from the penis with a resulting impotence [8-11]. The TA, being composed mainly of collagen fibers, is relatively noncompliant. It occludes the penile venous outflow through compression of the subtunical venular plexus and the perforating emissary veins passing through it. The integrity of the fibroelastic tissue of the TA and CC apparently plays a significant role in the erectile process [12].

### A concept on the pathogenesis of venogenic ED

Venogenic ED is considered to result from an improperly functioning occlusion mechanism [13,14]. Investigations lay stress on the role of the TA in the venous occlusion mechanism of the penis during erection [3,8,9,14]. In the current study, the TA collagen was found degenerated and atrophic and this probably leads to subluxation and *floppiness *of the TA as a tube surrounding the penile corporal tissue. It appears that the subluxated TA, during erection, fails to effect compression of both the subtunical venular plexus and the emissary veins passing through it. Failure of occlusion of the penile venous outflow presumably leads to venous leakage during erection and seems to explain the ED in these patients.

Furthermore, under normal physiologic conditions, the TA is responsible for morphologically shaping the penis during erection. The collagenous structure gives the TA a textile nature which firmly supports the penile architecture during penile tumescence. This TA textile nature, with its circularly and longitudinally oriented collagen fibers lends the TA an adaptability to adjust its length and breadth according to the penile status, whether flaccid or erected. Due to their inelasticity, the collagen fibers limit excessive tunical stretch during penile tumescence. This mechanism prevents tunical subluxation and *floppiness *which may result from repeated penile tumescence which occurs during the sexual life. Meanwhile, an atrophic subluxated TA would not only disrupt the "venous-leak proof" effect of the TA but disturb the solidity of the erected penis during the process of vaginal penetration and thrusting.

It may be argued that the TA changes are the result of venous leakage. However, this does not seem to be the case since the TA rigidity is claimed to act for prevention of venous lelakage during erection [8,9,13,14]. Therefore, although it appears inappropriate that venous leakage should lead to tunical subluxation and *floppiness*, this possibility cannot be excluded.

Investigators reported a significant decrease in elastic fibers in the TA of impotent patients in comparison to a control group [15]. A reduction of the TA elastic fibers is likely to produce disorders in the arrangement of collagen fibers [16,17]. As a matter of fact, electron microscopy revealed severe myopathic, fibrotic changes in the penile tissue [18].

The possible etiology of the atrophic subluxated TA needs to be discussed. The TA consists of collagen and thus may be involved in the pathology of collagen diseases. However, none of the patients had any of these conditions. Aging may affect the TA integrity, but the studied subjects were middle-aged. The history and investigative results of the patients have shown that they had no relevant pathological conditions nor were under drug regimens that may cause ED. However, a recent study has shown that androgen administered to patients with ED due to VED *has *improved erection in some, *but not in all *patients [19]. The investigators suggested that penile tissue remodeling is the mechanism *that is presumably *responsible for correction of the VED by androgen treatment. In view of these factual aspects, we hypothesize that collagenous degeneration and atrophy could be a primary pathologic condition affecting the TA.

A limitation of the study is that the cohort of the patients is not homogeneous for their characteristics. It was difficult to convince healthy subjects to have a biopsy taken from their penile TA. Therefore we seized the opportunity to take the TA biopsy from healthy subjects with normal erection during operative interference; this selection did not effect the results.

## Conclusion

The study has shown that during erection, the ICP of patients with VED was significantly lower than that of the controls. Furthermore, the collagen fibers of the TA exhibited degenerative and atrophic changes that presumably lead to TA subluxation and *floppiness*. These TA changes seem to explain the cause of the lowered ICP of the patients during erection which apparently results from loss of the TA veno-occlusive mechanism. The cause of atrophic changes and subluxation of the TA needs to be studied.

## Abbreviations

VED, venogenic erectile dysfunction; CC, corpora cavernosa; TA, tunica albuginea ICP, intracorporal pressure;

## Competing interests

The author(s) declare that they have no competing interests.

## Authors' contributions

AS carried out the study design, data collection, statistical analysis, data interpretation and preparation of manuscript. IA participated in data collection and analysis and literature search. OE participated in data collection, statistical analysis and literature search. AAS participated in data collection and preparation of the manuscript. All authors read and approved the final manuscript.

## Pre-publication history

The pre-publication history for this paper can be accessed here:


